# Body Image, Food Consumption and Physical Activity Associated With Weight Control Behaviours in Adolescents

**DOI:** 10.1111/apa.70543

**Published:** 2026-04-09

**Authors:** Leandro André Vieira Olsson, Giancarlo Bazarele Machado Bruno, Felipe Mendes Delpino, Elza Daniel de Mello, Clécio Homrich da Silva

**Affiliations:** ^1^ Postgraduate Program in Child and Adolescent Health Universidade Federal Do Rio Grande Do Sul Porto Alegre Rio Grande do Sul Brazil; ^2^ Federal Institute of Santa Catarina Florianópolis Santa Catarina Brazil; ^3^ Postgraduate Program in Nursing Universidade Federal de Pelotas Pelotas Rio Grande do Sul Brazil

**Keywords:** adolescents, body dissatisfaction, eating disorders, physical activity, weight control behaviours

## Abstract

**Aim:**

Body dissatisfaction and unhealthy weight control behaviours are common during adolescence. We examined associations between body image, diet, physical activity, and risk behaviours for weight control among high school students.

**Methods:**

This cross‐sectional study included 802 adolescents from five municipalities in southern Brazil, including 795 aged 14–19 years and seven aged 20 years. Data were collected between May and June 2022 using self‐administered questionnaires based on the National School Health Survey. Risk behaviours were defined as vomiting, laxative use, or use of weight‐loss products in the previous 30 days. Associations were analysed using Poisson regression with robust variance, adjusted for age, sex, maternal education, and socioeconomic status.

**Results:**

The sample included 802 adolescents, 50.0% male, with a mean age of 16.7 years (range 14–20). The prevalence of risk behaviours was 4.8%. Body dissatisfaction was associated with a higher likelihood of these behaviours (adjusted prevalence ratio 3.53, 95% confidence interval 1.65–7.53), with stronger effects among females (4.59, 1.33–15.78). No significant associations were found for diet or physical activity.

**Conclusions:**

Body dissatisfaction was associated with harmful weight control behaviours in adolescents and may support early identification and prevention strategies.

## Summary

- Body dissatisfaction and unhealthy weight control behaviours are common during adolescence, but evidence from Brazilian adolescents remains limited.
- Body dissatisfaction was associated with a higher likelihood of risk behaviours for weight control, especially among females, while no associations were observed for diet or physical activity.
- Early identification of body dissatisfaction may support targeted prevention strategies in school and clinical settings.

## 1 Introduction

Body dissatisfaction is a common concern during adolescence due to physical and psychological changes and has been associated with the development of unhealthy weight control behaviours [1, 2, 3, 4]. These behaviours include practices such as restrictive dieting, vomiting, and use of weight‐loss products and may increase the risk of eating disorders.

Body dissatisfaction has been reported more frequently during adolescence than at other stages of life and is more common among girls than boys [5, 6]. Among adolescents with overweight, there is a higher likelihood of adopting unhealthy eating behaviours, excessive concern about weight, and restrictive practices, which may further increase dissatisfaction with body image [7]. Previous studies have shown that body dissatisfaction and concern about weight gain are associated with higher levels of unhealthy weight control behaviours and an increased risk of eating disorders [8, 9].

Weight‐related concerns combined with unhealthy dietary patterns have also been identified as risk factors for overweight and eating disorders in adolescents [10]. In females, body dissatisfaction is typically associated with a desire to be thinner, whereas in males it is more often related to muscularity and body fat [11, 12]. These findings suggest that body dissatisfaction affects both sexes, although in different ways.

Despite the relevance of this topic, evidence from Brazil remains limited, particularly among adolescents from smaller municipalities and non‐metropolitan settings. This gap is important because adolescence is a critical period for the development of behaviours that may have long‐term health consequences. Therefore, this study aimed to examine whether body image, food consumption, and physical activity were associated with risk behaviours for weight control among high school students.

## 2 Methods

### 2.1 Study Design and Participants

This cross‐sectional study was conducted among high school students enrolled in the Federal Institute Farroupilha, a public institution in southern Brazil. Participants were recruited from campuses in five municipalities in the interior of Rio Grande do Sul, each with fewer than 100 000 inhabitants. Data were collected between May and June 2022 using self‐administered electronic questionnaires completed during scheduled school activities. Students aged 14–20 years were eligible. Seven participants were aged 20 years and were retained because they were enrolled in the same school environment as younger students and represented < 1% of the sample.

### 2.2 Data Collection

Data were collected using questionnaires based on the National School Health Survey, a widely used instrument for assessing health behaviours in adolescents. The questionnaire was adapted for online administration and completed through a secure platform.

### 2.3 Variables

Risk behaviours for weight control were defined as the report of at least one of the following behaviours in the previous 30 days: vomiting or use of laxatives to lose or prevent weight gain, or use of medications or other products for weight loss without medical supervision. These behaviours were self‐reported and were not used to establish a clinical diagnosis.

The main exposure variables were body satisfaction, food consumption, and physical activity. Food consumption was summarised using a composite score based on six items assessing frequency of healthy and unhealthy foods in the previous seven days. Unhealthy items were reverse‐coded so that higher scores indicated healthier patterns. The total score was standardised from 0 to 10 and categorised as predominantly unhealthy (< 3), intermediate (3–7), or predominantly healthy (≥ 8). Physical activity was assessed as total time in the previous seven days and categorised as < 150 min, 150–299 min, or ≥ 300 min per week.

### 2.4 Statistical Analysis

The required sample size was estimated at 722 participants to detect differences in risk behaviours, assuming 80% power and a 5% significance level. An additional 10% was included to account for potential losses, resulting in a target of 804 participants. Continuous variables were described using means and standard deviations, and categorical variables as counts and percentages. Group differences were assessed using the chi‐square test. Associations were analysed using Poisson regression with robust variance to estimate prevalence ratios and 95% confidence intervals, adjusted for age, sex, maternal education, and socioeconomic status. A *p* value < 0.05 was considered statistically significant. Analyses were conducted using the Statistical Package for the Social Sciences, version 22.0.

### 2.5 Ethics

The study was approved by the Research Ethics Committee of the Federal Institute Farroupilha and the National Research Ethics Commission (approval number 50315221.7.0000.5574). Written informed consent was obtained from participants aged 18 years or older, and assent with parental consent for those under 18 years. This study was reported in accordance with the Strengthening the Reporting of Observational Studies in Epidemiology guidelines [13].

## 3 Results

A total of 850 students were invited to participate. After excluding 48 non‐respondents, the final sample comprised 802 adolescents, 50.0% male, with a mean age of 16.7 ± 1.2 years (range 14–20), including seven participants aged 20 years. Risk behaviours for weight control were reported by 4.8% of adolescents, with 3.1% reporting vomiting or laxative use and 2.4% reporting use of medications or other products for weight loss. The definitions and categorisation of study variables are described in Table 1.

**TABLE 1:** Description of the variables studied in a sample of high school students aged 14–20 at the Instituto Federal Farroupilha—RS (2022).

Variables	Description	Responses/categories
Risk behaviour for weight control	In the last 30 days, have you vomited or taken laxatives to lose weight or avoid gaining weight?	Yes; no
	In the last 30 days, have you taken any medication, formula or other product to lose weight without medical supervision?	
Body satisfaction	How do you feel about your body?	Satisfied: combination of the categories satisfied and very satisfied; Indifferent; Dissatisfied: combination of the categories very dissatisfied, dissatisfied
Food consumption	In the last 7 days, on how many days did you eat beans?	Responses: I haven't eaten the food in the last 7 days; 1 day; 2 days; 3 days; 4 days; 5 days; 6 days; or every day. A score was compiled from the answers and three categories were created: Predominantly unhealthy; Intermediate; Predominantly healthy.
	In the last 7 days, on how many days did you eat at least one type of vegetable other than potatoes or cassava?	
	In the last 7 days, on how many days did you eat fresh fruit or fruit salad?	
	In the last 7 days, on how many days have you eaten sweet treats such as candies, confectionery, chocolates, chewing gum, sweets and others?	
	In the last 7 days, on how many days did you drink soda?	
	In the last 7 days, on how many days did you eat at snack bars, hot dog stands, pizzerias, fast food, etc.?	
Physical activity	Active commuting to school:	Based on the answers, a score was created consisting of the sum of the time (min) of all the physical activities carried out in the last 7 days, classified into three categories: ≤ 149 min; 150–299 min; ≥ 300 min
	In the last 7 days, on how many days did you walk or cycle to school?	
	How long do you spend walking or cycling to school?	
	In the last 7 days, on how many days did you walk or cycle home from school?	
	How long do you spend walking or cycling home from school?	
	Curricular physical activity:	
	In the last 7 days, how many days have you had PE lessons at school?	
	In the last 7 days, how much time per day have you done physical activity or practiced sport during PE lessons at school? Do not take into account the time spent on theoretical activities in the classroom	
	Extra‐curricular physical activity:	
	How many days in the last 7 days, excluding school PE lessons, did you do any physical activity?	
	How long a day did these activities last?	

Overall, 48.4% of adolescents reported being satisfied or very satisfied with their body image, while 33.0% reported dissatisfaction. Among males, 58.8% were satisfied and 23.5% dissatisfied, compared with 38.2% and 42.4% among females, respectively. Regarding food consumption, 25.0% had a predominantly healthy diet (mean score 7.89 ± 0.67), while 3.5% had a predominantly unhealthy diet (mean score 2.47 ± 0.45), with most classified as intermediate (mean score 5.49 ± 0.67). Physical activity levels were generally low, with 50.2% reporting < 150 min per week. Further descriptive characteristics are presented in Table 2.

**TABLE 2:** Frequency of the variables studied in the sample of high school adolescents from Instituto Farroupilha, RS, 2022.

Variables	Total, *n* (%)	Male, *n* (%)	Female, *n* (%)
Gender	802	401	401
Age in years	801	400	401
14–16	367 (45.8)	182 (45.5)	185 (46.1)
17–19	427 (53.3)	214 (53.5)	213 (53.1)
20	7 (0.9)	4 (1.0)	3 (0.7)
Weight control with vomiting and the use of laxatives	800	399	401
Yes	25 (3.1)	10 (2.5)	15 (3.7)
No	775 (96.9)	389 (97.5)	386 (96.3)
Weight loss with the use of medicines and formulas	800	399	401
Yes	19 (2.4)	9 (2.3)	10 (2.5)
No	781 (97.6)	390 (97.7)	391 (97.5)
Risk behaviour for weight loss	800	399	401
Yes	38 (4.8)	16 (4.0)	22 (5.5)
No	762 (95.2)	383 (96.0)	379 (94.5)
Body satisfaction	801	400	401
Very dissatisfied/dissatisfied	264 (33.0)	94 (23.5)	170 (42.4)
Indifferent	149 (18.6)	71 (17.7)	78 (19.4)
Satisfied/very satisfied	388 (48.4)	235 (58.8)	153 (38.2)
Food consumption	802	401	401
Predominantly unhealthy	28 (3.5)	12 (3.0)	16 (4.0)
Intermediate	573 (71.5)	296 (73.8)	277 (69.1)
Predominantly healthy	201 (25.0)	93 (23.2)	108 (26.9)
Physical activity (min/week)	797	399	398
≤ 149	400 (50.2)	170 (42.6)	230 (57.8)
150–299	205 (25.7)	106 (26.6)	99 (24.9)
≥ 300	192 (24.1)	123 (30.8)	69 (17.3)

Body dissatisfaction was associated with a higher likelihood of risk behaviours for weight control (*p* = 0.001). After adjustment, adolescents with body dissatisfaction were more likely to report these behaviours than those satisfied (prevalence ratio 3.53, 95% confidence interval 1.65–7.53). The association remained significant for males (prevalence ratio 3.04, 95% confidence interval 1.10–8.41) and was stronger among females (prevalence ratio 4.59, 95% confidence interval 1.33–15.78). No significant associations were observed between risk behaviours and food consumption (*p* = 0.947) or physical activity (*p* = 0.702), as shown in Table 3.

**TABLE 3:** Prevalence and prevalence ratio of risk behaviour for weight control, by gender, with the variables of body satisfaction, food consumption and physical activity, in a sample of high school students, aged 14 to 20, from the Instituto Federal Farroupilha (2022).

Variables	Prev. (%)	Crude analysis			Adjusted analysis		
		Prevalence ratio	Confidence interval 95%	*p*	Prevalence ratio	Confidence interval 95%	*p*
Body satisfaction							
Both sexes							
Very dissatisfied/dissatisfied	9.1	3.51	1.68–7.33	0.001	3.53	1.65–7.53	0.001
Indifferent	2.7	1.04	0.33–3.31	0.949	0.80	0.22–2.93	0.739
Satisfied/very satisfied	2.6	1			1		
Male							
Very dissatisfied/dissatisfied	8.5	2.85	1.03–7.85	0.043	3.04	1.10–8.41	0.032
Indifferent	1.4	0.47	0.06–3.83	0.481	0.48	0.06–3.91	0.494
Satisfied/very satisfied	3.0	1			1		
Female							
Very dissatisfied/dissatisfied	9.4	4.80	1.40–16.47	0.013	4.59	1.33–15.78	0.016
Indifferent	3.8	1.96	0.40–9.72	0.409	1.28	0.21–7.68	0.790
Satisfied/very satisfied	2.0	1			1		
Food consumption							
Both sexes							
Predominantly unhealthy	3.6	0.72	0.09–5.61	0.752	0.73	0.09–5.72	0.762
Intermediate	4.7	0.95	0.46–1.96	0.887	0.93	0.45–1.94	0.853
Predominantly healthy	5.0	1			1		
Male							
Predominantly unhealthy	0.0	—	—	—	—	—	—
Intermediate	4.8	2.21	0.50–9.74	0.293	2.34	0.53–10.33	0.263
Predominantly healthy	2.2	1			1		
Female							
Predominantly unhealthy	6.3	0.84	0.11–6.75	0.873	1.03	0.13–8.35	0.982
Intermediate	4.7	0.63	0.26–1.53	0.310	0.61	0.25–1.50	0.277
Predominantly healthy	7.4	1			1		
Physical activity (min/week)							
Both sexes							
≤ 149	5.0	1			1		
150–299	3.9	0.78	0.34–1.77	0.550	0.76	0.33–1.73	0.505
≥ 300	5.2	1.04	0.49–2.22	0.921	0.93	0.42–2.09	0.869
Male							
≤ 149	3.6	1			1		
150–299	3.8	1.06	0.30–3.74	0.932	1.03	0.29–3.67	0.961
≥ 300	4.9	1.37	0.44–4.24	0.589	1.30	0.42–4.06	0.649
Female							
≤ 149	6.1	1			1		
150–299	4.0	0.66	0.22–2.02	0.470	0.64	0.21–1.98	0.441
≥ 300	5.8	0.95	0.31–2.89	0.931	0.67	0.19–2.34	0.525

## 4 Discussion

This study examined the relationship between body dissatisfaction, food consumption and physical activity and risk behaviours for weight control among adolescents. The main finding was that body dissatisfaction was strongly associated with these behaviours, particularly among females, while no meaningful associations were observed for diet or physical activity. These results highlight the importance of body image as a central factor influencing unhealthy weight control practices during adolescence.

The prevalence of risk behaviours observed in this study was lower than that reported in national and international studies. Previous analyses based on the National School Health Survey in Brazil and studies from other countries have consistently reported higher prevalence, especially among females [14, 15, 16, 17]. This difference may be related to the characteristics of the present sample, which included adolescents from smaller municipalities, where social context, access to weight loss products, and patterns of reporting may differ from larger urban settings.

The association between body dissatisfaction and risk behaviours for weight control is consistent with previous evidence. Studies conducted in Brazil and internationally have shown that dissatisfaction with body image is associated with a higher likelihood of engaging in unhealthy weight control practices and disordered eating behaviours [8, 18, 19, 20]. In the present study, the magnitude of this association was relatively high, particularly among females, suggesting that body dissatisfaction may play a more prominent role in certain contexts. Together, these findings reinforce the idea that psychosocial factors are key drivers of these behaviours.

No significant associations were observed between risk behaviours and food consumption or physical activity. This is in line with previous studies reporting weak or inconsistent relationships between these variables [19, 20]. One possible explanation is that behaviours such as vomiting or laxative use are more closely linked to body image concerns and psychological distress than to routine lifestyle patterns. In addition, the use of composite measures for diet and broad categories for physical activity may have limited the ability to detect more subtle associations.

A strength of this study is the use of simple and direct measures that can be easily applied in both clinical and school settings. The findings show that straightforward questions about body satisfaction can help identify adolescents at risk of harmful weight control behaviours. In addition, the inclusion of adolescents from smaller municipalities contributes to addressing an important gap in the literature, which has predominantly focused on large urban populations.

However, we are not free of limitations. The cross‐sectional design does not allow causal inferences, and the results should be interpreted as associations. The sample was limited to a single public educational network, which may restrict generalisability. All variables were self‐reported, and no clinical or psychological assessments were performed. The outcome combined behaviours with different levels of severity, which may mask differences between specific practices. Finally, the relatively low prevalence of risk behaviours may have reduced the statistical power to detect weaker associations.

## 5 Conclusion

In this study, risk behaviours for weight control were relatively uncommon, but were clearly associated with body dissatisfaction, particularly among female adolescents. No meaningful associations were observed with food consumption or physical activity. These findings suggest that body image plays a central role in unhealthy weight control practices and highlight the importance of early identification of adolescents at risk in both clinical and school settings.

## Author Contributions

L.A.V.O., F.M.D., E.D.M., and C.H.S. conceptualised the study. L.A.V.O. and G.B.M.B. developed the methodology. L.A.V.O. and F.M.D. conducted data curation and analysis. L.A.V.O. and F.M.D. drafted the manuscript. All authors critically revised the manuscript and approved the final version. L.A.V.O. and F.M.D. had full access to the data and take responsibility for its integrity and accuracy.

## Funding

The authors have nothing to report.

## Conflicts of Interest

The authors declare no conflicts of interest.

## Supporting information


**Data S1:** apa70543‐sup‐0001‐DataS1.doc.

## Data Availability

De‐identified datasets and analysis scripts are available from the corresponding author upon reasonable request and subject to institutional ethics approval. Data collection instruments are available as Supporting Information.
